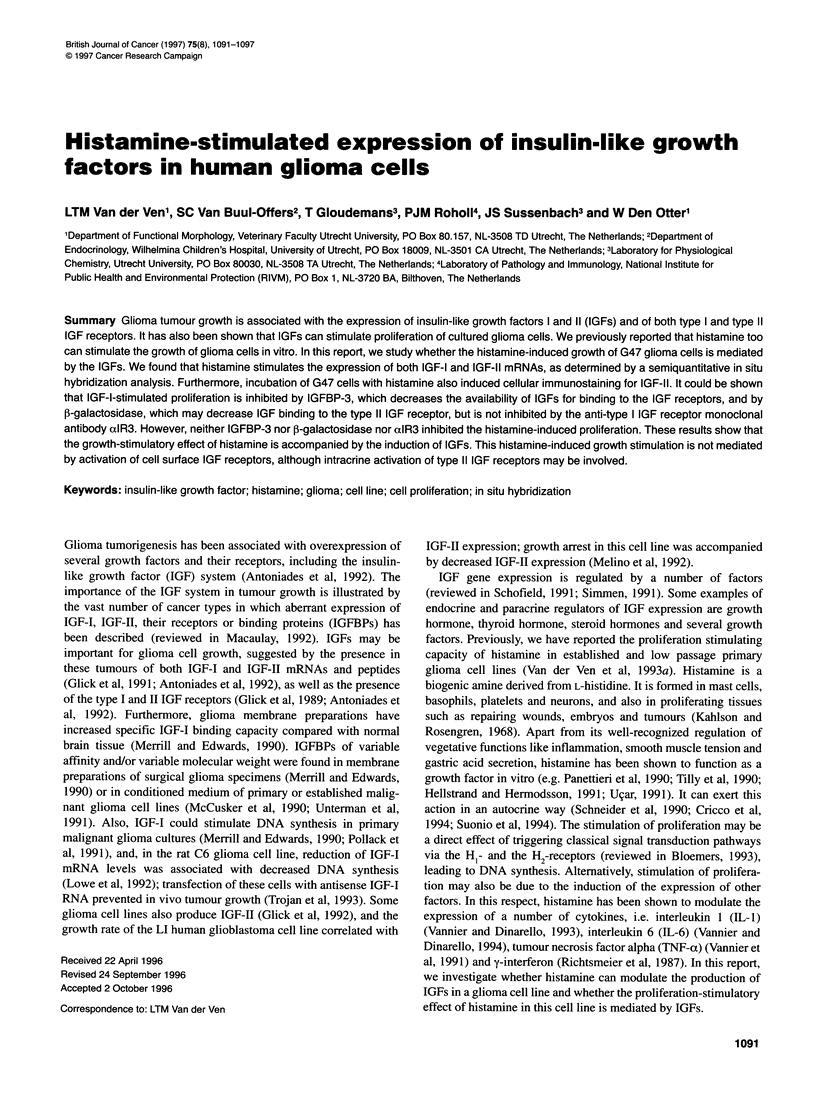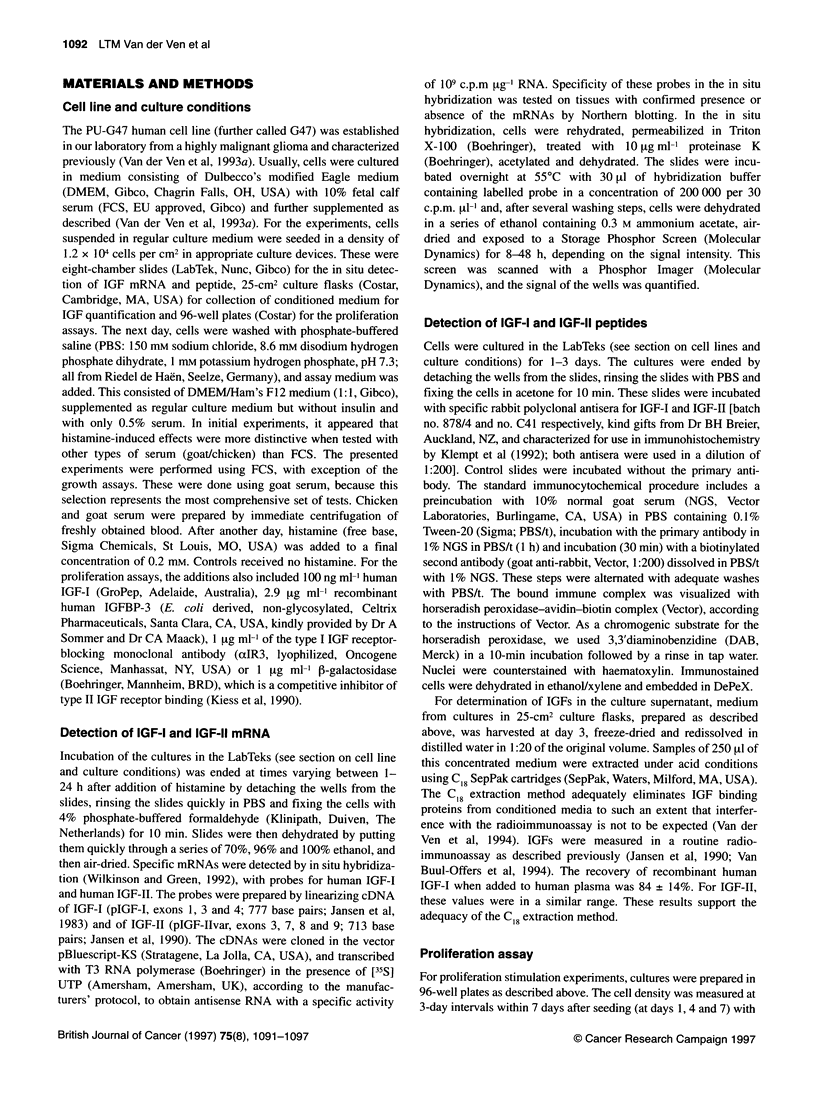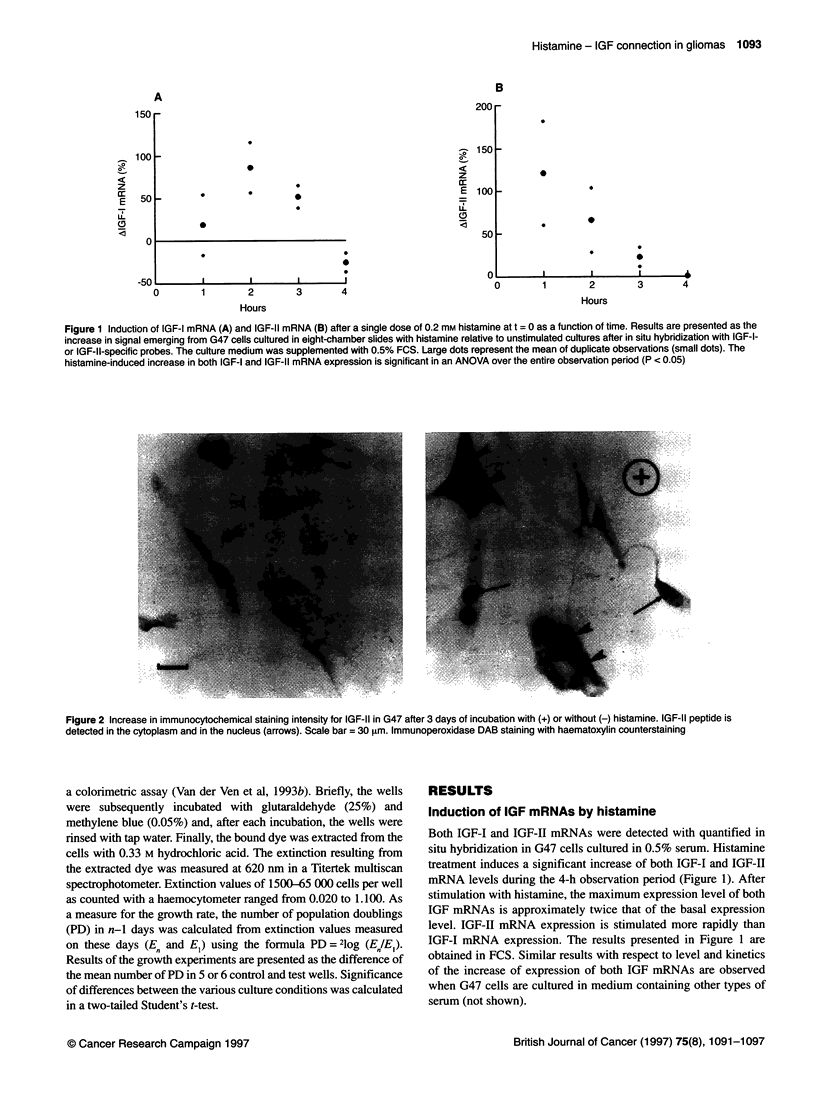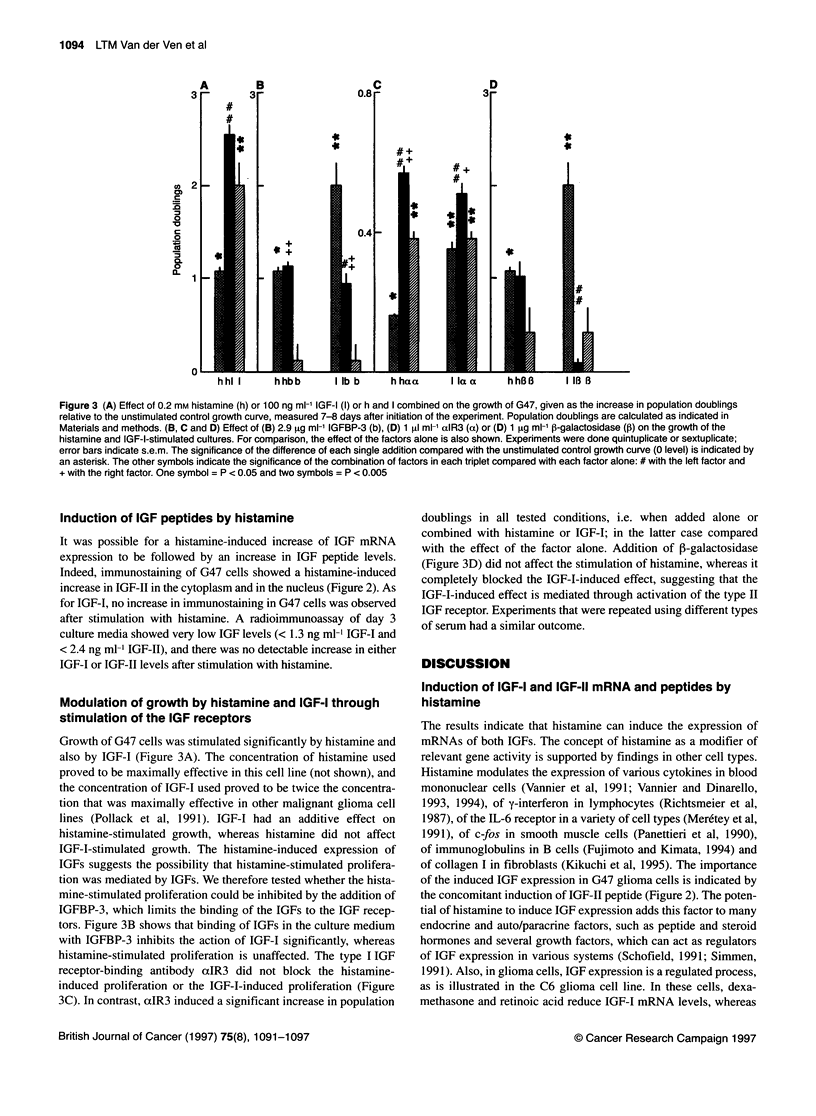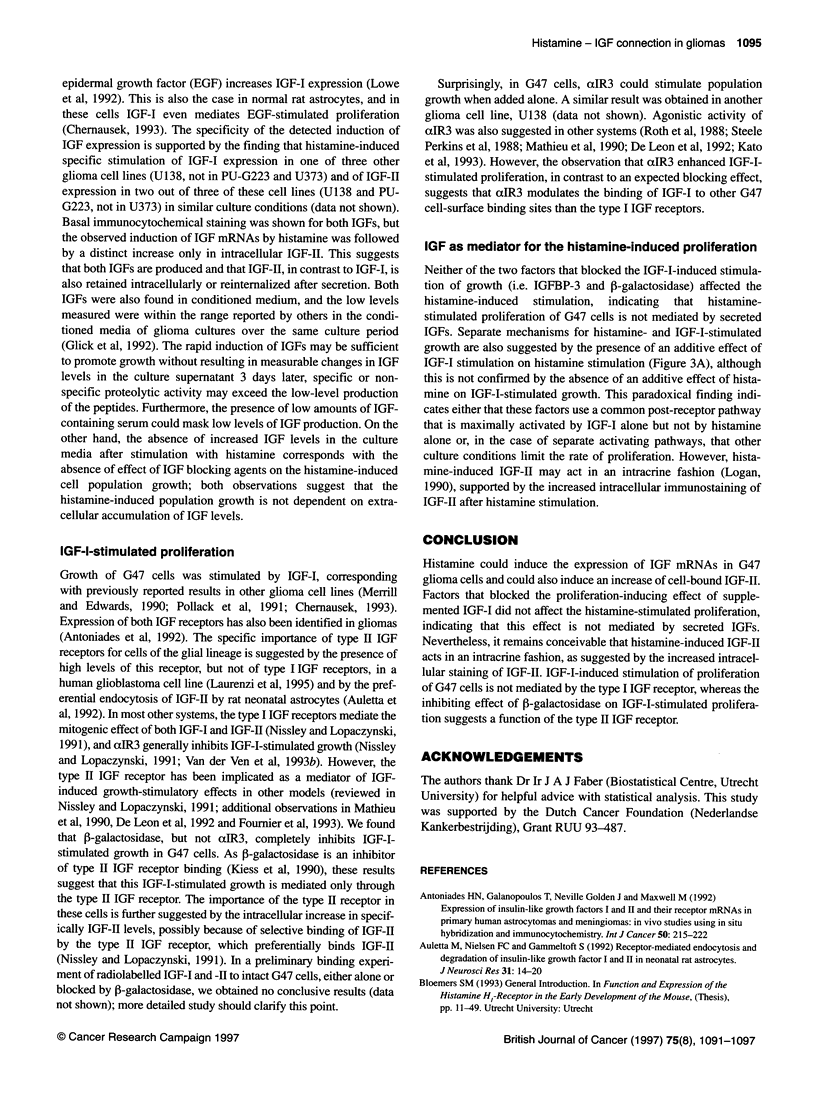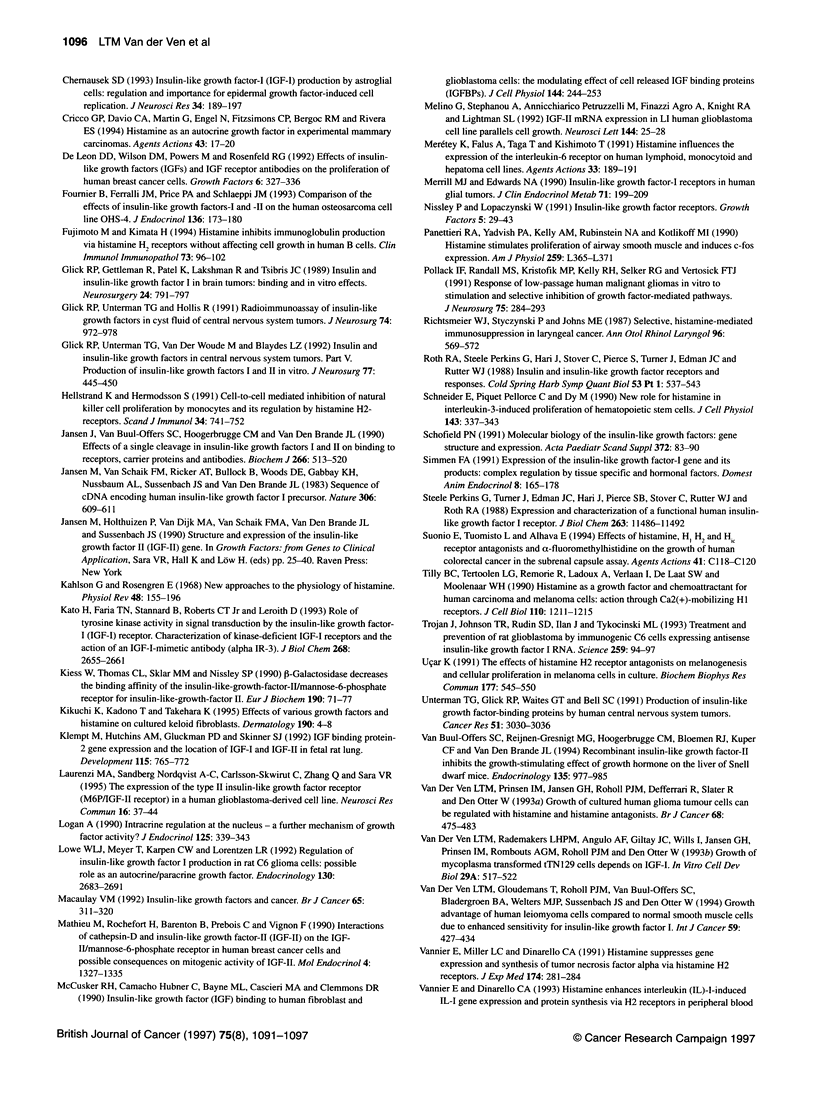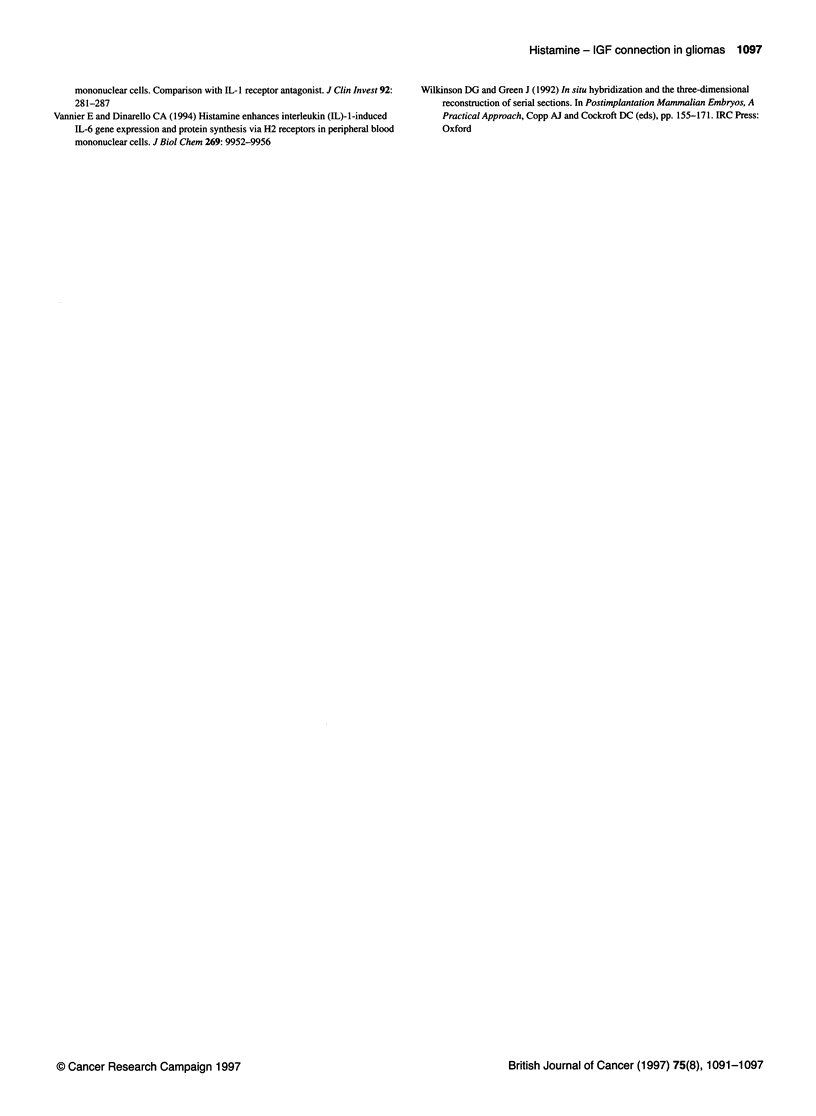# Histamine-stimulated expression of insulin-like growth factors in human glioma cells.

**DOI:** 10.1038/bjc.1997.189

**Published:** 1997

**Authors:** L. T. Van der Ven, S. C. Van Buul-Offers, T. Gloudemans, P. J. Roholl, J. S. Sussenbach, W. Den Otter

**Affiliations:** Department of Functional Morphology, Veterinary Faculty Utrecht University, The Netherlands.

## Abstract

**Images:**


British Journal of Cancer (1997) 75(8), 1091–1097
© 1997 Cancer Research Campaign

### Histamine-stimulated expression of insulin-like growth factors in human glioma cells

**LTM Van der Ven[1], SC Van Buul-Offers[2], T Gloudemans[3], PJM Roholl[4], JS Sussenbach[3] and W Den Otter[1]**

[1]Department of Functional Morphology, Veterinary Faculty Utrecht University, PO Box 80.157, NL-3508 TD Utrecht, The Netherlands; [2]Department of Endocrinology, Wilhelmina Children's Hospital, University of Utrecht, PO Box 18009, NL-3501 CA Utrecht, The Netherlands; [3]Laboratory for Physiological Chemistry, Utrecht University, PO Box 80030, NL-3508 TA Utrecht, The Netherlands; [4]Laboratory of Pathology and Immunology, National Institute for Public Health and Environmental Protection (RIVM), PO Box 1, NL-3720 BA, Bilthoven, The Netherlands

**Summary** Glioma tumour growth is associated with the expression of insulin-like growth factors I and II (IGFs) and of both type I and type II IGF receptors. It has also been shown that IGFs can stimulate proliferation of cultured glioma cells. We previously reported that histamine too can stimulate the growth of glioma cells in vitro. In this report, we study whether the histamine-induced growth of G47 glioma cells is mediated by the IGFs. We found that histamine stimulates the expression of both IGF-I and IGF-II mRNAs, as determined by a semiquantitative in situ hybridization analysis. Furthermore, incubation of G47 cells with histamine also induced cellular immunostaining for IGF-II. It could be shown that IGF-I-stimulated proliferation is inhibited by IGFBP-3, which decreases the availability of IGFs for binding to the IGF receptors, and by β-galactosidase, which may decrease IGF binding to the type II IGF receptor, but is not inhibited by the anti-type I IGF receptor monoclonal antibody αIR3. However, neither IGFBP-3 nor β-galactosidase nor αIR3 inhibited the histamine-induced proliferation. These results show that the growth-stimulatory effect of histamine is accompanied by the induction of IGFs. This histamine-induced growth stimulation is not mediated by activation of cell surface IGF receptors, although intracrine activation of type II IGF receptors may be involved.

**Keywords:** insulin-like growth factor; histamine; glioma; cell line; cell proliferation; in situ hybridization

Glioma tumorigenesis has been associated with overexpression of several growth factors and their receptors, including the insulin-like growth factor (IGF) system (Antoniades et al, 1992). The importance of the IGF system in tumour growth is illustrated by the vast number of cancer types in which aberrant expression of IGF-I, IGF-II, their receptors or binding proteins (IGFBPs) has been described (reviewed in Macaulay, 1992). IGFs may be important for glioma cell growth, suggested by the presence in these tumours of both IGF-I and IGF-II mRNAs and peptides (Glick et al, 1991; Antoniades et al, 1992), as well as the presence of the type I and II IGF receptors (Glick et al, 1989; Antoniades et al, 1992). Furthermore, glioma membrane preparations have increased specific IGF-I binding capacity compared with normal brain tissue (Merrill and Edwards, 1990). IGFBPs of variable affinity and/or variable molecular weight were found in membrane preparations of surgical glioma specimens (Merrill and Edwards, 1990) or in conditioned medium of primary or established malignant glioma cell lines (McCusker et al, 1990; Unterman et al, 1991). Also, IGF-I could stimulate DNA synthesis in primary malignant glioma cultures (Merrill and Edwards, 1990; Pollack et al, 1991), and, in the rat C6 glioma cell line, reduction of IGF-I mRNA levels was associated with decreased DNA synthesis (Lowe et al, 1992); transfection of these cells with antisense IGF-I RNA prevented in vivo tumour growth (Trojan et al, 1993). Some glioma cell lines also produce IGF-II (Glick et al, 1992), and the growth rate of the LI human glioblastoma cell line correlated with IGF-II expression; growth arrest in this cell line was accompanied by decreased IGF-II expression (Melino et al, 1992).

Received 22 April 1996
Revised 24 September 1996
Accepted 2 October 1996

Correspondence to: LTM Van der Ven

IGF gene expression is regulated by a number of factors (reviewed in Schofield, 1991; Simmen, 1991). Some examples of endocrine and paracrine regulators of IGF expression are growth hormone, thyroid hormone, steroid hormones and several growth factors. Previously, we have reported the proliferation stimulating capacity of histamine in established and low passage primary glioma cell lines (Van der Ven et al, 1993*a*). Histamine is a biogenic amine derived from L-histidine. It is formed in mast cells, basophils, platelets and neurons, and also in proliferating tissues such as repairing wounds, embryos and tumours (Kahlson and Rosengren, 1968). Apart from its well-recognized regulation of vegetative functions like inflammation, smooth muscle tension and gastric acid secretion, histamine has been shown to function as a growth factor in vitro (e.g. Panettieri et al, 1990; Tilly et al, 1990; Hellstrand and Hermodsson, 1991; Uçar, 1991). It can exert this action in an autocrine way (Schneider et al, 1990; Cricco et al, 1994; Suonio et al, 1994). The stimulation of proliferation may be a direct effect of triggering classical signal transduction pathways via the $H_1$- and the $H_2$-receptors (reviewed in Bloemers, 1993), leading to DNA synthesis. Alternatively, stimulation of proliferation may also be due to the induction of the expression of other factors. In this respect, histamine has been shown to modulate the expression of a number of cytokines, i.e. interleukin 1 (IL-1) (Vannier and Dinarello, 1993), interleukin 6 (IL-6) (Vannier and Dinarello, 1994), tumour necrosis factor alpha (TNF-α) (Vannier et al, 1991) and γ-interferon (Richtsmeier et al, 1987). In this report, we investigate whether histamine can modulate the production of IGFs in a glioma cell line and whether the proliferation-stimulatory effect of histamine in this cell line is mediated by IGFs.

### MATERIALS AND METHODS

### Cell line and culture conditions

The PU-G47 human cell line (further called G47) was established in our laboratory from a highly malignant glioma and characterized previously (Van der Ven et al, 1993*a*). Usually, cells were cultured in medium consisting of Dulbecco's modified Eagle medium (DMEM, Gibco, Chagrin Falls, OH, USA) with 10% fetal calf serum (FCS, EU approved, Gibco) and further supplemented as described (Van der Ven et al, 1993*a*). For the experiments, cells suspended in regular culture medium were seeded in a density of $1.2 \times 10^4$ cells per $cm^2$ in appropriate culture devices. These were eight-chamber slides (LabTek, Nunc, Gibco) for the in situ detection of IGF mRNA and peptide, 25-$cm^2$ culture flasks (Costar, Cambridge, MA, USA) for collection of conditioned medium for IGF quantification and 96-well plates (Costar) for the proliferation assays. The next day, cells were washed with phosphate-buffered saline (PBS: 150 mM sodium chloride, 8.6 mM disodium hydrogen phosphate dihydrate, 1 mM potassium hydrogen phosphate, pH 7.3; all from Riedel de Haën, Seelze, Germany), and assay medium was added. This consisted of DMEM/Ham's F12 medium (1:1, Gibco), supplemented as regular culture medium but without insulin and with only 0.5% serum. In initial experiments, it appeared that histamine-induced effects were more distinctive when tested with other types of serum (goat/chicken) than FCS. The presented experiments were performed using FCS, with exception of the growth assays. These were done using goat serum, because this selection represents the most comprehensive set of tests. Chicken and goat serum were prepared by immediate centrifugation of freshly obtained blood. After another day, histamine (free base, Sigma Chemicals, St Louis, MO, USA) was added to a final concentration of 0.2 mM. Controls received no histamine. For the proliferation assays, the additions also included 100 ng $ml^{-1}$ human IGF-I (GroPep, Adelaide, Australia), 2.9 μg $ml^{-1}$ recombinant human IGFBP-3 (*E. coli* derived, non-glycosylated, Celtrix Pharmaceuticals, Santa Clara, CA, USA, kindly provided by Dr A Sommer and Dr CA Maack), 1 μg $ml^{-1}$ of the type I IGF receptor-blocking monoclonal antibody (αIR3, lyophilized, Oncogene Science, Manhassat, NY, USA) or 1 μg $ml^{-1}$ β-galactosidase (Boehringer, Mannheim, BRD), which is a competitive inhibitor of type II IGF receptor binding (Kiess et al, 1990).

### Detection of IGF-I and IGF-II mRNA

Incubation of the cultures in the LabTeks (see section on cell line and culture conditions) was ended at times varying between 1–24 h after addition of histamine by detaching the wells from the slides, rinsing the slides quickly in PBS and fixing the cells with 4% phosphate-buffered formaldehyde (Klinipath, Duiven, The Netherlands) for 10 min. Slides were then dehydrated by putting them quickly through a series of 70%, 96% and 100% ethanol, and then air-dried. Specific mRNAs were detected by in situ hybridization (Wilkinson and Green, 1992), with probes for human IGF-I and human IGF-II. The probes were prepared by linearizing cDNA of IGF-I (pIGF-I, exons 1, 3 and 4; 777 base pairs; Jansen et al, 1983) and of IGF-II (pIGF-IIvar, exons 3, 7, 8 and 9; 713 base pairs; Jansen et al, 1990). The cDNAs were cloned in the vector pBluescript-KS (Stratagene, La Jolla, CA, USA), and transcribed with T3 RNA polymerase (Boehringer) in the presence of [$^{35}$S] UTP (Amersham, Amersham, UK), according to the manufacturers' protocol, to obtain antisense RNA with a specific activity of $10^9$ c.p.m μ$g^{-1}$ RNA. Specificity of these probes in the in situ hybridization was tested on tissues with confirmed presence or absence of the mRNAs by Northern blotting. In the in situ hybridization, cells were rehydrated, permeabilized in Triton X-100 (Boehringer), treated with 10 μg $ml^{-1}$ proteinase K (Boehringer), acetylated and dehydrated. The slides were incubated overnight at 55°C with 30 μl of hybridization buffer containing labelled probe in a concentration of 200 000 per 30 c.p.m. μ$l^{-1}$ and, after several washing steps, cells were dehydrated in a series of ethanol containing 0.3 M ammonium acetate, air-dried and exposed to a Storage Phosphor Screen (Molecular Dynamics) for 8–48 h, depending on the signal intensity. This screen was scanned with a Phosphor Imager (Molecular Dynamics), and the signal of the wells was quantified.

### Detection of IGF-I and IGF-II peptides

Cells were cultured in the LabTeks (see section on cell lines and culture conditions) for 1–3 days. The cultures were ended by detaching the wells from the slides, rinsing the slides with PBS and fixing the cells in acetone for 10 min. These slides were incubated with specific rabbit polyclonal antisera for IGF-I and IGF-II [batch no. 878/4 and no. C41 respectively, kind gifts from Dr BH Breier, Auckland, NZ, and characterized for use in immunohistochemistry by Klempt et al (1992); both antisera were used in a dilution of 1:200]. Control slides were incubated without the primary antibody. The standard immunocytochemical procedure includes a preincubation with 10% normal goat serum (NGS, Vector Laboratories, Burlingame, CA, USA) in PBS containing 0.1% Tween-20 (Sigma; PBS/t), incubation with the primary antibody in 1% NGS in PBS/t (1 h) and incubation (30 min) with a biotinylated second antibody (goat anti-rabbit, Vector, 1:200) dissolved in PBS/t with 1% NGS. These steps were alternated with adequate washes with PBS/t. The bound immune complex was visualized with horseradish peroxidase–avidin–biotin complex (Vector), according to the instructions of Vector. As a chromogenic substrate for the horseradish peroxidase, we used 3,3'diaminobenzidine (DAB, Merck) in a 10-min incubation followed by a rinse in tap water. Nuclei were counterstained with haematoxylin. Immunostained cells were dehydrated in ethanol/xylene and embedded in DePeX.

For determination of IGFs in the culture supernatant, medium from cultures in 25-$cm^2$ culture flasks, prepared as described above, was harvested at day 3, freeze-dried and redissolved in distilled water in 1:20 of the original volume. Samples of 250 μl of this concentrated medium were extracted under acid conditions using $C_{18}$ SepPak cartridges (SepPak, Waters, Milford, MA, USA). The $C_{18}$ extraction method adequately eliminates IGF binding proteins from conditioned media to such an extent that interference with the radioimmunoassay is not to be expected (Van der Ven et al, 1994). IGFs were measured in a routine radioimmunoassay as described previously (Jansen et al, 1990; Van Buul-Offers et al, 1994). The recovery of recombinant human IGF-I when added to human plasma was 84 ± 14%. For IGF-II, these values were in a similar range. These results support the adequacy of the $C_{18}$ extraction method.

### Proliferation assay

For proliferation stimulation experiments, cultures were prepared in 96-well plates as described above. The cell density was measured at 3-day intervals within 7 days after seeding (at days 1, 4 and 7) with

© Cancer Research Campaign 1997

**Figure 1** Induction of IGF-I mRNA (**A**) and IGF-II mRNA (**B**) after a single dose of 0.2 mM histamine at t = 0 as a function of time. Results are presented as the increase in signal emerging from G47 cells cultured in eight-chamber slides with histamine relative to unstimulated cultures after in situ hybridization with IGF-I- or IGF-II-specific probes. The culture medium was supplemented with 0.5% FCS. Large dots represent the mean of duplicate observations (small dots). The histamine-induced increase in both IGF-I and IGF-II mRNA expression is significant in an ANOVA over the entire observation period ($P < 0.05$)

**Figure 2** Increase in immunocytochemical staining intensity for IGF-II in G47 after 3 days of incubation with (+) or without (–) histamine. IGF-II peptide is detected in the cytoplasm and in the nucleus (arrows). Scale bar = 30 μm. Immunoperoxidase DAB staining with haematoxylin counterstaining a colorimetric assay (Van der Ven et al, 1993*b*). Briefly, the wells were subsequently incubated with glutaraldehyde (25%) and methylene blue (0.05%) and, after each incubation, the wells were rinsed with tap water. Finally, the bound dye was extracted from the cells with 0.33 M hydrochloric acid. The extinction resulting from the extracted dye was measured at 620 nm in a Titertek multiscan spectrophotometer. Extinction values of 1500–65 000 cells per well as counted with a haemocytometer ranged from 0.020 to 1.100. As a measure for the growth rate, the number of population doublings (PD) in $n$–1 days was calculated from extinction values measured on these days ($E_n$ and $E_1$) using the formula PD = ${}^2\log(E_n/E_1)$. Results of the growth experiments are presented as the difference of the mean number of PD in 5 or 6 control and test wells. Significance of differences between the various culture conditions was calculated in a two-tailed Student's $t$-test.

### RESULTS

### Induction of IGF mRNAs by histamine

Both IGF-I and IGF-II mRNAs were detected with quantified in situ hybridization in G47 cells cultured in 0.5% serum. Histamine treatment induces a significant increase of both IGF-I and IGF-II mRNA levels during the 4-h observation period (Figure 1). After stimulation with histamine, the maximum expression level of both IGF mRNAs is approximately twice that of the basal expression level. IGF-II mRNA expression is stimulated more rapidly than IGF-I mRNA expression. The results presented in Figure 1 are obtained in FCS. Similar results with respect to level and kinetics of the increase of expression of both IGF mRNAs are observed when G47 cells are cultured in medium containing other types of serum (not shown).

© Cancer Research Campaign 1997

**Figure 3** (**A**) Effect of 0.2 mM histamine (h) or 100 ng $ml^{-1}$ IGF-I (I) or h and I combined on the growth of G47, given as the increase in population doublings relative to the unstimulated control growth curve, measured 7–8 days after initiation of the experiment. Population doublings are calculated as indicated in Materials and methods. (**B, C** and **D**) Effect of (**B**) 2.9 μg $ml^{-1}$ IGFBP-3 (b), (**D**) 1 μl $ml^{-1}$ αIR3 (α) or (**D**) 1 μg $ml^{-1}$ β-galactosidase (β) on the growth of the histamine and IGF-I-stimulated cultures. For comparison, the effect of the factors alone is also shown. Experiments were done quintuplicate or sextuplicate; error bars indicate s.e.m. The significance of the difference of each single addition compared with the unstimulated control growth curve (0 level) is indicated by an asterisk. The other symbols indicate the significance of the combination of factors in each triplet compared with each factor alone: # with the left factor and + with the right factor. One symbol = $P < 0.05$ and two symbols = $P < 0.005$

### Induction of IGF peptides by histamine

It was possible for a histamine-induced increase of IGF mRNA expression to be followed by an increase in IGF peptide levels. Indeed, immunostaining of G47 cells showed a histamine-induced increase in IGF-II in the cytoplasm and in the nucleus (Figure 2). As for IGF-I, no increase in immunostaining in G47 cells was observed after stimulation with histamine. A radioimmunoassay of day 3 culture media showed very low IGF levels (< 1.3 ng $ml^{-1}$ IGF-I and < 2.4 ng $ml^{-1}$ IGF-II), and there was no detectable increase in either IGF-I or IGF-II levels after stimulation with histamine.

### Modulation of growth by histamine and IGF-I through stimulation of the IGF receptors

Growth of G47 cells was stimulated significantly by histamine and also by IGF-I (Figure 3A). The concentration of histamine used proved to be maximally effective in this cell line (not shown), and the concentration of IGF-I used proved to be twice the concentration that was maximally effective in other malignant glioma cell lines (Pollack et al, 1991). IGF-I had an additive effect on histamine-stimulated growth, whereas histamine did not affect IGF-I-stimulated growth. The histamine-induced expression of IGFs suggests the possibility that histamine-stimulated proliferation was mediated by IGFs. We therefore tested whether the histamine-stimulated proliferation could be inhibited by the addition of IGFBP-3, which limits the binding of the IGFs to the IGF receptors. Figure 3B shows that binding of IGFs in the culture medium with IGFBP-3 inhibits the action of IGF-I significantly, whereas histamine-stimulated proliferation is unaffected. The type I IGF receptor-binding antibody αIR3 did not block the histamine-induced proliferation or the IGF-I-induced proliferation (Figure 3C). In contrast, αIR3 induced a significant increase in population doublings in all tested conditions, i.e. when added alone or combined with histamine or IGF-I; in the latter case compared with the effect of the factor alone. Addition of β-galactosidase (Figure 3D) did not affect the stimulation of histamine, whereas it completely blocked the IGF-I-induced effect, suggesting that the IGF-I-induced effect is mediated through activation of the type II IGF receptor. Experiments that were repeated using different types of serum had a similar outcome.

### DISCUSSION

### Induction of IGF-I and IGF-II mRNA and peptides by histamine

The results indicate that histamine can induce the expression of mRNAs of both IGFs. The concept of histamine as a modifier of relevant gene activity is supported by findings in other cell types. Histamine modulates the expression of various cytokines in blood mononuclear cells (Vannier et al, 1991; Vannier and Dinarello, 1993, 1994), of γ-interferon in lymphocytes (Richtsmeier et al, 1987), of the IL-6 receptor in a variety of cell types (Merétey et al, 1991), of c-*fos* in smooth muscle cells (Panettieri et al, 1990), of immunoglobulins in B cells (Fujimoto and Kimata, 1994) and of collagen I in fibroblasts (Kikuchi et al, 1995). The importance of the induced IGF expression in G47 glioma cells is indicated by the concomitant induction of IGF-II peptide (Figure 2). The potential of histamine to induce IGF expression adds this factor to many endocrine and auto/paracrine factors, such as peptide and steroid hormones and several growth factors, which can act as regulators of IGF expression in various systems (Schofield, 1991; Simmen, 1991). Also, in glioma cells, IGF expression is a regulated process, as is illustrated in the C6 glioma cell line. In these cells, dexamethasone and retinoic acid reduce IGF-I mRNA levels, whereas

© Cancer Research Campaign 1997

epidermal growth factor (EGF) increases IGF-I expression (Lowe et al, 1992). This is also the case in normal rat astrocytes, and in these cells IGF-I even mediates EGF-stimulated proliferation (Chernausek, 1993). The specificity of the detected induction of IGF expression is supported by the finding that histamine-induced specific stimulation of IGF-I expression in one of three other glioma cell lines (U138, not in PU-G223 and U373) and of IGF-II expression in two out of three of these cell lines (U138 and PU-G223, not in U373) in similar culture conditions (data not shown). Basal immunocytochemical staining was shown for both IGFs, but the observed induction of IGF mRNAs by histamine was followed by a distinct increase only in intracellular IGF-II. This suggests that both IGFs are produced and that IGF-II, in contrast to IGF-I, is also retained intracellularly or reinternalized after secretion. Both IGFs were also found in conditioned medium, and the low levels measured were within the range reported by others in the conditioned media of glioma cultures over the same culture period (Glick et al, 1992). The rapid induction of IGFs may be sufficient to promote growth without resulting in measurable changes in IGF levels in the culture supernatant 3 days later, specific or non-specific proteolytic activity may exceed the low-level production of the peptides. Furthermore, the presence of low amounts of IGF-containing serum could mask low levels of IGF production. On the other hand, the absence of increased IGF levels in the culture media after stimulation with histamine corresponds with the absence of effect of IGF blocking agents on the histamine-induced cell population growth; both observations suggest that the histamine-induced population growth is not dependent on extracellular accumulation of IGF levels.

### IGF-I-stimulated proliferation

Growth of G47 cells was stimulated by IGF-I, corresponding with previously reported results in other glioma cell lines (Merrill and Edwards, 1990; Pollack et al, 1991; Chernausek, 1993). Expression of both IGF receptors has also been identified in gliomas (Antoniades et al, 1992). The specific importance of type II IGF receptors for cells of the glial lineage is suggested by the presence of high levels of this receptor, but not of type I IGF receptors, in a human glioblastoma cell line (Laurenzi et al, 1995) and by the preferential endocytosis of IGF-II by rat neonatal astrocytes (Auletta et al, 1992). In most other systems, the type I IGF receptors mediate the mitogenic effect of both IGF-I and IGF-II (Nissley and Lopaczynski, 1991), and αIR3 generally inhibits IGF-I-stimulated growth (Nissley and Lopaczynski, 1991; Van der Ven et al, 1993*b*). However, the type II IGF receptor has been implicated as a mediator of IGF-induced growth-stimulatory effects in other models (reviewed in Nissley and Lopaczynski, 1991; additional observations in Mathieu et al, 1990, De Leon et al, 1992 and Fournier et al, 1993). We found that β-galactosidase, but not αIR3, completely inhibits IGF-I-stimulated growth in G47 cells. As β-galactosidase is an inhibitor of type II IGF receptor binding (Kiess et al, 1990), these results suggest that this IGF-I-stimulated growth is mediated only through the type II IGF receptor. The importance of the type II receptor in these cells is further suggested by the intracellular increase in specifically IGF-II levels, possibly because of selective binding of IGF-II by the type II IGF receptor, which preferentially binds IGF-II (Nissley and Lopaczynski, 1991). In a preliminary binding experiment of radiolabelled IGF-I and -II to intact G47 cells, either alone or blocked by β-galactosidase, we obtained no conclusive results (data not shown); more detailed study should clarify this point.

Surprisingly, in G47 cells, αIR3 could stimulate population growth when added alone. A similar result was obtained in another glioma cell line, U138 (data not shown). Agonistic activity of αIR3 was also suggested in other systems (Roth et al, 1988; Steele Perkins et al, 1988; Mathieu et al, 1990; De Leon et al, 1992; Kato et al, 1993). However, the observation that αIR3 enhanced IGF-I-stimulated proliferation, in contrast to an expected blocking effect, suggests that αIR3 modulates the binding of IGF-I to other G47 cell-surface binding sites than the type I IGF receptors.

### IGF as mediator for the histamine-induced proliferation

Neither of the two factors that blocked the IGF-I-induced stimulation of growth (i.e. IGFBP-3 and β-galactosidase) affected the histamine-induced stimulation, indicating that histamine-stimulated proliferation of G47 cells is not mediated by secreted IGFs. Separate mechanisms for histamine- and IGF-I-stimulated growth are also suggested by the presence of an additive effect of IGF-I stimulation on histamine stimulation (Figure 3A), although this is not confirmed by the absence of an additive effect of histamine on IGF-I-stimulated growth. This paradoxical finding indicates either that these factors use a common post-receptor pathway that is maximally activated by IGF-I alone but not by histamine alone or, in the case of separate activating pathways, that other culture conditions limit the rate of proliferation. However, histamine-induced IGF-II may act in an intracrine fashion (Logan, 1990), supported by the increased intracellular immunostaining of IGF-II after histamine stimulation.

### CONCLUSION

Histamine could induce the expression of IGF mRNAs in G47 glioma cells and could also induce an increase of cell-bound IGF-II. Factors that blocked the proliferation-inducing effect of supplemented IGF-I did not affect the histamine-stimulated proliferation, indicating that this effect is not mediated by secreted IGFs. Nevertheless, it remains conceivable that histamine-induced IGF-II acts in an intracrine fashion, as suggested by the increased intracellular staining of IGF-II. IGF-I-induced stimulation of proliferation of G47 cells is not mediated by the type I IGF receptor, whereas the inhibiting effect of β-galactosidase on IGF-I-stimulated proliferation suggests a function of the type II IGF receptor.

### ACKNOWLEDGEMENTS

The authors thank Dr Ir J A J Faber (Biostatistical Centre, Utrecht University) for helpful advice with statistical analysis. This study was supported by the Dutch Cancer Foundation (Nederlandse Kankerbestrijding), Grant RUU 93–487.

### REFERENCES

Antoniades HN, Galanopoulos T, Neville Golden J and Maxwell M (1992) Expression of insulin-like growth factors I and II and their receptor mRNAs in primary human astrocytomas and meningiomas: in vivo studies using in situ hybridization and immunocytochemistry. *Int J Cancer* **50**: 215–222

Auletta M, Nielsen FC and Gammeltoft S (1992) Receptor-mediated endocytosis and degradation of insulin-like growth factor I and II in neonatal rat astrocytes. *J Neurosci Res* **31**: 14–20

Bloemers SM (1993) General Introduction. In *Function and Expression of the Histamine $H_1$-Receptor in the Early Development of the Mouse*, (Thesis), pp. 11–49. Utrecht University: Utrecht

© Cancer Research Campaign 1997 

Chernausek SD (1993) Insulin-like growth factor-I (IGF-I) production by astroglial cells: regulation and importance for epidermal growth factor-induced cell replication. *J Neurosci Res* **34**: 189–197

Cricco GP, Davio CA, Martin G, Engel N, Fitzsimons CP, Bergoc RM and Rivera ES (1994) Histamine as an autocrine growth factor in experimental mammary carcinomas. *Agents Actions* **43**: 17–20

De Leon DD, Wilson DM, Powers M and Rosenfeld RG (1992) Effects of insulin-like growth factors (IGFs) and IGF receptor antibodies on the proliferation of human breast cancer cells. *Growth Factors* **6**: 327–336

Fournier B, Ferralli JM, Price PA and Schlaeppi JM (1993) Comparison of the effects of insulin-like growth factors-I and -II on the human osteosarcoma cell line OHS-4. *J Endocrinol* **136**: 173–180

Fujimoto M and Kimata H (1994) Histamine inhibits immunoglobulin production via histamine $H_2$ receptors without affecting cell growth in human B cells. *Clin Immunol Immunopathol* **73**: 96–102

Glick RP, Gettleman R, Patel K, Lakshman R and Tsibris JC (1989) Insulin and insulin-like growth factor I in brain tumors: binding and in vitro effects. *Neurosurgery* **24**: 791–797

Glick RP, Unterman TG and Hollis R (1991) Radioimmunoassay of insulin-like growth factors in cyst fluid of central nervous system tumors. *J Neurosurg* **74**: 972–978

Glick RP, Unterman TG, Van Der Woude M and Blaydes LZ (1992) Insulin and insulin-like growth factors in central nervous system tumors. Part V. Production of insulin-like growth factors I and II in vitro. *J Neurosurg* **77**: 445–450

Hellstrand K and Hermodsson S (1991) Cell-to-cell mediated inhibition of natural killer cell proliferation by monocytes and its regulation by histamine H2-receptors. *Scand J Immunol* **34**: 741–752

Jansen J, Van Buul-Offers SC, Hoogerbrugge CM and Van Den Brande JL (1990) Effects of a single cleavage in insulin-like growth factors I and II on binding to receptors, carrier proteins and antibodies. *Biochem J* **266**: 513–520

Jansen M, Van Schaik FM, Ricker AT, Bullock B, Woods DE, Gabbay KH, Nussbaum AL, Sussenbach JS and Van Den Brande JL (1983) Sequence of cDNA encoding human insulin-like growth factor I precursor. *Nature* **306**: 609–611

Jansen M, Holthuizen P, Van Dijk MA, Van Schaik FMA, Van Den Brande JL and Sussenbach JS (1990) Structure and expression of the insulin-like growth factor II (IGF-II) gene. In *Growth Factors: from Genes to Clinical Application*, Sara VR, Hall K and Löw H. (eds) pp. 25–40. Raven Press: New York

Kahlson G and Rosengren E (1968) New approaches to the physiology of histamine. *Physiol Rev* **48**: 155–196

Kato H, Faria TN, Stannard B, Roberts CT Jr and Leroith D (1993) Role of tyrosine kinase activity in signal transduction by the insulin-like growth factor-I (IGF-I) receptor. Characterization of kinase-deficient IGF-I receptors and the action of an IGF-I-mimetic antibody (alpha IR-3). *J Biol Chem* **268**: 2655–2661

Kiess W, Thomas CL, Sklar MM and Nissley SP (1990) β-Galactosidase decreases the binding affinity of the insulin-like-growth-factor-II/mannose-6-phosphate receptor for insulin-like-growth-factor II. *Eur J Biochem* **190**: 71–77

Kikuchi K, Kadono T and Takehara K (1995) Effects of various growth factors and histamine on cultured keloid fibroblasts. *Dermatology* **190**: 4–8

Klempt M, Hutchins AM, Gluckman PD and Skinner SJ (1992) IGF binding protein-2 gene expression and the location of IGF-I and IGF-II in fetal rat lung. *Development* **115**: 765–772

Laurenzi MA, Sandberg Nordqvist A-C, Carlsson-Skwirut C, Zhang Q and Sara VR (1995) The expression of the type II insulin-like growth factor receptor (M6P/IGF-II receptor) in a human glioblastoma-derived cell line. *Neurosci Res Commun* **16**: 37–44

Logan A (1990) Intracrine regulation at the nucleus – a further mechanism of growth factor activity? *J Endocrinol* **125**: 339–343

Lowe WLJ, Meyer T, Karpen CW and Lorentzen LR (1992) Regulation of insulin-like growth factor I production in rat C6 glioma cells: possible role as an autocrine/paracrine growth factor. *Endocrinology* **130**: 2683–2691

Macaulay VM (1992) Insulin-like growth factors and cancer. *Br J Cancer* **65**: 311–320

Mathieu M, Rochefort H, Barenton B, Prebois C and Vignon F (1990) Interactions of cathepsin-D and insulin-like growth factor-II (IGF-II) on the IGF-II/mannose-6-phosphate receptor in human breast cancer cells and possible consequences on mitogenic activity of IGF-II. *Mol Endocrinol* **4**: 1327–1335

McCusker RH, Camacho Hubner C, Bayne ML, Cascieri MA and Clemmons DR (1990) Insulin-like growth factor (IGF) binding to human fibroblast and glioblastoma cells: the modulating effect of cell released IGF binding proteins (IGFBPs). *J Cell Physiol* **144**: 244–253

Melino G, Stephanou A, Annicchiarico Petruzzelli M, Finazzi Agro A, Knight RA and Lightman SL (1992) IGF-II mRNA expression in LI human glioblastoma cell line parallels cell growth. *Neurosci Lett* **144**: 25–28

Merétey K, Falus A, Taga T and Kishimoto T (1991) Histamine influences the expression of the interleukin-6 receptor on human lymphoid, monocytoid and hepatoma cell lines. *Agents Actions* **33**: 189–191

Merrill MJ and Edwards NA (1990) Insulin-like growth factor-I receptors in human glial tumors. *J Clin Endocrinol Metab* **71**: 199–209

Nissley P and Lopaczynski W (1991) Insulin-like growth factor receptors. *Growth Factors* **5**: 29–43

Panettieri RA, Yadvish PA, Kelly AM, Rubinstein NA and Kotlikoff MI (1990) Histamine stimulates proliferation of airway smooth muscle and induces c-fos expression. *Am J Physiol* **259**: L365–L371

Pollack IF, Randall MS, Kristofik MP, Kelly RH, Selker RG and Vertosick FTJ (1991) Response of low-passage human malignant gliomas in vitro to stimulation and selective inhibition of growth factor-mediated pathways. *J Neurosurg* **75**: 284–293

Richtsmeier WJ, Styczynski P and Johns ME (1987) Selective, histamine-mediated immunosuppression in laryngeal cancer. *Ann Otol Rhinol Laryngol* **96**: 569–572

Roth RA, Steele Perkins G, Hari J, Stover C, Pierce S, Turner J, Edman JC and Rutter WJ (1988) Insulin and insulin-like growth factor receptors and responses. *Cold Spring Harb Symp Quant Biol* **53 Pt 1**: 537–543

Schneider E, Piquet Pellorce C and Dy M (1990) New role for histamine in interleukin-3-induced proliferation of hematopoietic stem cells. *J Cell Physiol* **143**: 337–343

Schofield PN (1991) Molecular biology of the insulin-like growth factors: gene structure and expression. *Acta Paediatr Scand Suppl* **372**: 83–90

Simmen FA (1991) Expression of the insulin-like growth factor-I gene and its products: complex regulation by tissue specific and hormonal factors. *Domest Anim Endocrinol* **8**: 165–178

Steele Perkins G, Turner J, Edman JC, Hari J, Pierce SB, Stover C, Rutter WJ and Roth RA (1988) Expression and characterization of a functional human insulin-like growth factor I receptor. *J Biol Chem* **263**: 11486–11492

Suonio E, Tuomisto L and Alhava E (1994) Effects of histamine, $H_1$ $H_2$ and $H_{ic}$ receptor antagonists and α-fluoromethylhistidine on the growth of human colorectal cancer in the subrenal capsule assay. *Agents Actions* **41**: C118–C120

Tilly BC, Tertoolen LG, Remorie R, Ladoux A, Verlaan I, De Laat SW and Moolenaar WH (1990) Histamine as a growth factor and chemoattractant for human carcinoma and melanoma cells: action through Ca2(+)-mobilizing H1 receptors. *J Cell Biol* **110**: 1211–1215

Trojan J, Johnson TR, Rudin SD, Ilan J and Tykocinski ML (1993) Treatment and prevention of rat glioblastoma by immunogenic C6 cells expressing antisense insulin-like growth factor I RNA. *Science* **259**: 94–97

Uçar K (1991) The effects of histamine H2 receptor antagonists on melanogenesis and cellular proliferation in melanoma cells in culture. *Biochem Biophys Res Commun* **177**: 545–550

Unterman TG, Glick RP, Waites GT and Bell SC (1991) Production of insulin-like growth factor-binding proteins by human central nervous system tumors. *Cancer Res* **51**: 3030–3036

Van Buul-Offers SC, Reijnen-Gresnigt MG, Hoogerbrugge CM, Bloemen RJ, Kuper CF and Van Den Brande JL (1994) Recombinant insulin-like growth factor-II inhibits the growth-stimulating effect of growth hormone on the liver of Snell dwarf mice. *Endocrinology* **135**: 977–985

Van Der Ven LTM, Prinsen IM, Jansen GH, Roholl PJM, Defferrari R, Slater R and Den Otter W (1993*a*) Growth of cultured human glioma tumour cells can be regulated with histamine and histamine antagonists. *Br J Cancer* **68**: 475–483

Van Der Ven LTM, Rademakers LHPM, Angulo AF, Giltay JC, Wills I, Jansen GH, Prinsen IM, Rombouts AGM, Roholl PJM and Den Otter W (1993*b*) Growth of mycoplasma transformed tTN129 cells depends on IGF-I. *In Vitro Cell Dev Biol* **29A**: 517–522

Van Der Ven LTM, Gloudemans T, Roholl PJM, Van Buul-Offers SC, Bladergroen BA, Welters MJP, Sussenbach JS and Den Otter W (1994) Growth advantage of human leiomyoma cells compared to normal smooth muscle cells due to enhanced sensitivity for insulin-like growth factor I. *Int J Cancer* **59**: 427–434

Vannier E, Miller LC and Dinarello CA (1991) Histamine suppresses gene expression and synthesis of tumor necrosis factor alpha via histamine H2 receptors. *J Exp Med* **174**: 281–284

Vannier E and Dinarello CA (1993) Histamine enhances interleukin (IL)-I-induced IL-I gene expression and protein synthesis via H2 receptors in peripheral blood

© Cancer Research Campaign 1997

mononuclear cells. Comparison with IL-1 receptor antagonist. *J Clin Invest* **92**: 281–287

Vannier E and Dinarello CA (1994) Histamine enhances interleukin (IL)-1-induced IL-6 gene expression and protein synthesis via H2 receptors in peripheral blood mononuclear cells. *J Biol Chem* **269**: 9952–9956

Wilkinson DG and Green J (1992) *In situ* hybridization and the three-dimensional reconstruction of serial sections. In *Postimplantation Mammalian Embryos, A Practical Approach*, Copp AJ and Cockroft DC (eds), pp. 155–171. IRC Press: Oxford

© Cancer Research Campaign 1997